# Novel clinicopathological and molecular characterization of metanephric adenoma: a study of 28 cases

**DOI:** 10.1186/s13000-018-0732-x

**Published:** 2018-08-16

**Authors:** Ying Ding, Cong Wang, Xuejie Li, Yangyang Jiang, Ping Mei, Wenbin Huang, Guoxin Song, Jinsong Wang, Guoqiang Ping, Ran Hu, Chen Miao, Xiao He, Gang Chen, Hai Li, Yan Zhu, Zhihong Zhang

**Affiliations:** 1 Department of Pathology, The First Affiliated Hospital of Nanjing Medical University, 300 Guangzhou Road, Nanjing, 210029 China; 2 Department of Pathology, The First Affiliated Hospital, School of Medicine, Zhejiang Unversity, Hangzhou, 310003 China; 3 Department of Pathology, Shenzhen People’s Hospital, Shenzhen, 518020 China; 4 Department of Pathology, Guangdong General Hospital/Guangdong Academy of Medical Sciences, Guangzhou, 510000 China; 5 Department of Pathology, Nanjing First Hospital, Nanjing, 210000 China

**Keywords:** Metanephric adenoma, Next-generation sequencing (NGS), BRAF V600E mutation, Mitogen-activated protein kinase (MAPK) pathway

## Abstract

**Background:**

Metanephric adenoma is a rare, benign renal neoplasm with occasional misdiagnosis. However, its molecular characterization is not fully understood.

**Methods:**

In this study, we use the hybrid capture-based Next-Generation Sequencing to sequence a panel of 295 well-established oncogene or tumor suppressor genes in 28 cases of MA patients in China. Novel clinicopathological markers associated with the mitogen-activated protein kinase (MAPK) pathway in metanephric adenoma were detected by immunohistochemistry.

**Results:**

It was found that except for *BRAF* (22/28) mutations (c.1799 T > A, p.V600E), *NF1* (6/28), *NOTCH1* (5/28), *SPEN* (5/28), *AKT2* (4/28), *APC* (4/28), *ATRX* (3/28), and *ETV4* (3/28) mutations could also be detected. Meanwhile, a novel and rare gene fusion of *STARD9-BRAF*, *CUX1-BRAF*, and *LOC100507389-BRAF* was detected in one MA patient. In addition, although MEK phosphorylation was normally activated, the phosphorylation level of ERK was low in metanephric adenoma cases. Highly expressed p16 and DUSP6 may have contributed to these results, which maintained MA as a benign renal tumor.

**Conclusions:**

This study provides novel molecular and pathological markers for metanephric adenoma, which could improve its diagnosis and increase the understanding of its pathologic mechanism.

**Electronic supplementary material:**

The online version of this article (10.1186/s13000-018-0732-x) contains supplementary material, which is available to authorized users.

## Background

Metanephric adenoma (MA) is a rare, benign renal neoplasm, as it exhibits a low proliferation rate and favorable outcome [1]. MA patients are usually asymptomatic, incidentally discovered, and occur across different age groups with a female predominance (female: male = 2: 1) [1, 2]. Histologically, MA is comprised of primitive metanephric tubular epithelial cells, which are arranged tightly as small acini structures [3, 4]. Papillary, tubular, and glomeruloid growth patterns can be identified in most MA cases [4]. Tumor cells have round nuclei, scant cytoplasm, extremely low mitotic activity, and rare necrosis [5].

However, it is difficult to distinguish MA from other malignant renal cell carcinoma using imaging studies [1]. Besides, the histopathological diagnosis of MA could be challenging [3, 6]. There is similar morphology between MA and other renal tumors such as epithelial-predominant nephroblastoma (Wilms tumor) and the solid variant of type 1 papillary renal cell carcinoma (PRCC), both of which demonstrate aggressive behavior. These similar morphologies seriously influence clinical diagnosis and therapy [6, 7]. Therefore, in some questionable cases, especially core needle biopsy samples, immunohistochemistry and fluorescence in situ hybridization (FISH) analysis may be useful in the identification of MA. Udager et al. showed that positive immunostaining of WT1 and CD57, negative of CK7 and AMACR could be a characteristic of MA [6]. Meanwhile, it was found that MA lacks copy number variants of chromosomes 7, 17, and Y, which are typical in type 1 PRCC [8].

Recently, genetic analysis has revealed the novel molecular characteristics of MA. It was demonstrated that missense mutation of BRAF V600E could be detected in approximately 90% of this kidney tumor subtype [3]. In addition, other somatic mutations at *BRAF* exon 15, including a V600D missense mutation and a V600D and K601 L double mutation were also reported [6]. Oncogene *BRAF* encodes a serine/threonine kinase protein, which could be activated by RAS kinase and subsequently phosphorylate MEK kinase to involve the mitogen-activated protein kinase (MAPK) signaling pathway, thereby regulating cell division and differentiation [9]. However, the BRAF V600E mutation could improve BRAF kinase activity and sustain the activation of downstream kinase MEK, which occurs in some human malignancies, such as melanoma, papillary thyroid carcinoma, colonic adenocarcinoma, pulmonary cancer, Langerhans cell histiocytosis, and pleomorphic xanthoastrocytomas to stimulate tumor growth [10, 11]. However, in some BRAF V600E-mutated indolent neoplasms such as melanocytic nevi and MA, the MAPK cascades are activated as well as that in malignant tumors, but their progression proceeds slowly [12]. Its underlying mechanism has not been fully understood. Increased tumor suppressor p16 (INK4α) expression in these BRAF V600E-mutated indolent or benign neoplasms may partly explain this phenomenon, which causes cell cycle arrest and senescence [13, 14]. Nevertheless, as BRAF V600E mutation is quite rare or even absent in other common renal tumors, it could be used as a molecular marker for the detection of MA.

In this study, we generated a retrospective cohort of 28 MA cases from multiple pathology centers in China to identify more histopathological and molecular features of this rare tumor within the Asian population. Using gene analysis based on Next-Generation Sequencing (NGS), the genetic profiles of MA were described. Meanwhile, novel histopathological markers in MA were also investigated to further explore its possible development patterns.

## Methods

### Patients and samples

Thirty-six cases originally diagnosed as MA were collected from the surgical pathology files of nine participating institutions based in China between 2012 and 2016. Slides stained with hematoxylin and eosin (H&E) from all original cases were reviewed by two expert pathologists who were blinded to both the clinical and the genetic results. To compare to morphologic mimics, 15 cases of solid variant papillary renal cell carcinoma and 15 cases of epithelial-predominant nephroblastoma were also analyzed. A total of twenty-eight MA cases were confirmed. Clinical parameters, pathological data, and follow-up information of these twenty-eight MA cases were systematically collected. The study was approved by the Ethics Committee of the First Affiliated Hospital of Nanjing Medical University (No. 2016-SRFA-011, the ethics committee did not require additional informed consent to be obtained for this retrospective study).

### DNA preparations and NGS analysis

Formalin fixed paraffin-embedded (FFPE) tissue blocks were used for DNA isolation using the QIAamp DNA FFPE Tissue Kit (QIAGEN). Briefly, 4 μm sections were made: the tumor area and the adjacent normal pericarcinous tissue were divided on H&E-stained slides, and tumor content > 70% were separated for subsequent DNA isolation. DNA concentration and fragmentation were examined to ensure DNA quality.

For NGS analysis, DNA samples were profiled using a commercially available, capture-based targeted sequencing panel (Burning Rock Biotech Ltd., Guangzhou, China), targeting 295 genes and spanning 1.5 Mb of human genomic regions, including 65 drug targets, 107 well-established oncogene or tumor suppressor genes, and 12 tumor-relevant signaling pathway kinases (Additional file 1: Table S1). All genes were referred to COSMIC, OncoKB and ClinVar database [15]. Indexed samples were sequenced on the Miseq500 Desktop Sequencer instrument (Illumina, Inc., CA, US) with pair-end reads. Sequencing data were mapped to the human genome (hg19) using BWA aligner 0.7.10. Local alignment optimization, variant calling, and annotation were performed using GATK 3.2, MuTect, and VarScan. Variants at loci with a depth of less than 100 were filtered out using the VarScan fpfilter pipeline. A minimum of 5 supporting reads were needed for INDELs and 8 supporting reads were needed for SNV calling. According to the ExAC, 1000 Genomes, dbSNP and ESP6500SI-V2 databases, variants with population frequencies of over 0.1% were grouped as germline mutation. Remaining somatic variants were annotated with ANNOVAR and SnpEff v3.6. DNA translocation analysis was performed using both Tophat2 and Factera 1.4.3.

### Gene mutations in different renal carcinomas from cBioPortal for Cancer genomics database

To compare the gene mutation spectrum of MA and other renal tumors, especially Wilms tumor and PRCC, which have morphological similarities with MA, we collected the gene mutation data from cBioPortal for Cancer Genomics database [16, 17]. In cBioPortal database, 499 cases of clear cell RCC were originally from TCGA database (https://tcga-data.nci.nih.gov), 102 cases of Wilms tumor were from TARGET data (https://ocg.cancer.gov/programs/target/data-matrix), 293 cases of PRCC were from TCGA database.

### Amplification refractory mutation system and sanger sequencing

Instances of the BRAF V600E mutation were confirmed using the ADx-ARMS®*BRAF* Mutation Assay Kit (AmoyDx, China) by ARMS (Amplification Refractory Mutation System) and Scorpions technologies. Using flanking sequence-specific primers (forward: 5’-TTTGTGAATACTGGGAACTATGAAA-3′, reverse: 5’-TCATCCTAACACATTTCAAGCC-3′) and HotStarTaq DNA polymerase (Qiagen), *BRAF* exon 15 was amplified by PCR. The PCR products were detected with bidirectional Sanger sequencing using the Prism® 3100 Genetic Analyzer (ABI, CA, US). The resulting chromatograms were analyzed with Chromas software, version Pro 2.23 and compared with a reference sequence for *BRAF* exon 15 (NM_004333.4). Commercially available BRAF V600E-mutated or wild-type human genomic DNA were utilized as positive and negative controls respectively.

### Immunohistochemistry

Tissue sections were deparaffinized and rehydrated, and antigen was retrieved by citrate buffer in a pressure cooker at 125 °C for 4 min. The primary antibodies that were used are listed in Table 1. After antigen retrieval, sections were incubated with different primary antibodies at 4 °C overnight, then stained by Dako EnVision+ Systerm and DAB chromogen (Dako) incubation following their protocols. Positive and negative controls were used for each antigen.

**Table 1:** Antibodies used

Name of antibody	Protein target	Manufacturer	Catalog	Dilution used
CK7	Cytokeratin 7	Fuzhou Maixin Biotech	Kit-0021	1:1
P504S	alpha-methylacyl-CoA racemase (AMACR), P504S	Fuzhou Maixin Biotech	RMA-0546	1:1
WT-1	Wilms tumor 1	Fuzhou Maixin Biotech	MAB-0678	1:1
CD57	human natural killer-1	Fuzhou Maixin Biotech	MAB-0257	1:1
P53	p53	Fuzhou Maixin Biotech	MAB-0674	1:1
BCL2	B-cell lymphoma 2	Fuzhou Maixin Biotech	RMA-0660	1:1
CCND1	Cyclin-D1	Abcam	ab16663	1:200
P16	P16INK4A	Fuzhou Maixin Biotech	MAB-0673	1:1
p-ERK	phosphorylated Thr202/Tyr204-p44/42 MAPK	Cell Signaling Tech	4370	1:100
ERK	total p44/42 MAPK	Cell Signaling Tech	4695	1:100
p-MEK	phosphorylated MEK1/2 (Ser221)	Cell Signaling Tech	2338	1:100
MEK	total MEK1/2	Cell Signaling Tech	4694	1:100
DUSP4/MKP-2	Dual specificity protein phosphatase 4/MAP kinase phosphatase-2	Abcam	ab216576	1:50
DUSP6/MKP-3	Dual specificity protein phosphatase 6/MAP kinase phosphatase-3	Abcam	ab54940	1:50

The extent of immunohistochemical staining was double-blind evaluated by two expert pathologists. Tumor cells that showed less staining (0%) were considered negative, while tumor cells with 1 to 100% staining were positive. Over 50% of the tumor cells stained positivity were scored as diffuse and strong (3+); 26 to 50% were evaluated as intermediate and moderate (2+); and between 1 and 25% were scored as focal and weak (1+).

## Results

### Clinicopathologic features of a retrospective MA cohort

Thirty-six cases originally diagnosed as MA from nine pathology centers in China were analyzed in this study. After subsequent diagnosis, it was found that eight cases were misdiagnosed: two cases were reclassified as solid variant papillary renal cell carcinoma, one was epithelial-predominant nephroblastoma, one was tubulocystic carcinoma, one was renal mucinous tubular and spindle cell carcinoma (MTSCC), one was juxtaglomerular cell tumor (reninoma), one was renal oncocytoma, and one was renal solitary fibrous tumor. In which, tubulocystic carcinoma, MTSCC, reninoma, renal oncocytoma and renal solitary fibrous tumor have different morphological characterizes with MA, which were excluded after double-blinded review by two expert pathologists from our department. The remainders, two cases of PRCC and one case of Wilms tumor, which exerted similar morphological features with MA, were further identified by immunohistochemical study and *BRAF* mutations detection using qPCR. Thus, twenty-eight confirmed MA cases were available in this study (female: male = 18: 10); the age of patients at diagnosis ranged from 12 to 80 years (median age = 39 year). Sixteen of the patients developed MA in the right kidney, whereas 12 of the patients had MA in the left kidney. All MA tissues were obtained from partial or total nephrectomy specimens. The largest dimension of tumors ranged from 2 to 7 cm (median size = 3.1 cm). Except for ‘not available’ cases (*n* = 3), all the MA patients (*n* = 25) have survived until now. Patient and tumor characteristics are summarized in Additional file 2: Table S2.

**Fig. 1:**
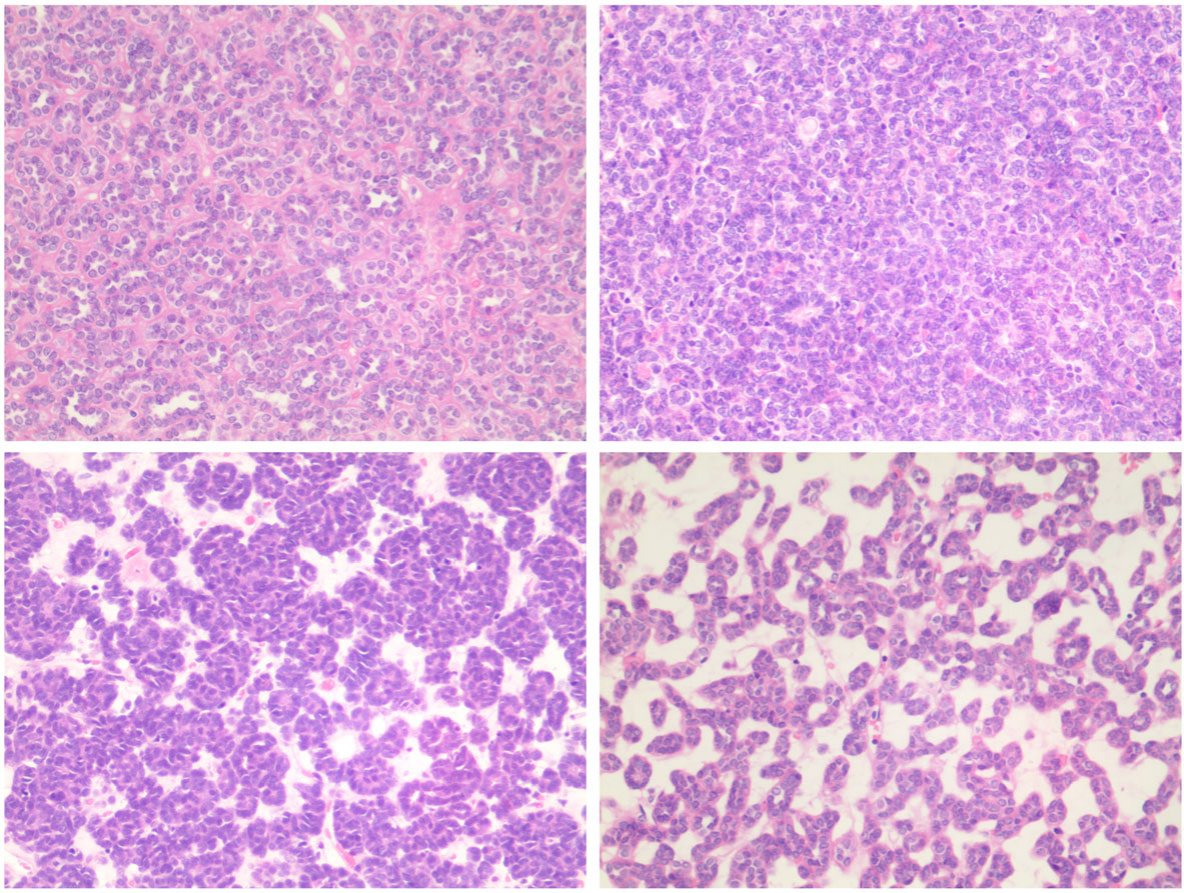
Representative images of H&E stataining of MA, 200X

Microscopically, all MAs exhibit small uniform epithelial cells. These tumors were found with scant cytoplasm, dark nuclei without nucleoli, no mitotic figure was found. Most cells were arranged in small acinar structures, and simple tubules, papillary, glomeruloid growth patterns, and solid structures could be also identified (Fig. 1).

### Deep-sequencing reveals novel mutations in MA

**Fig. 2:**
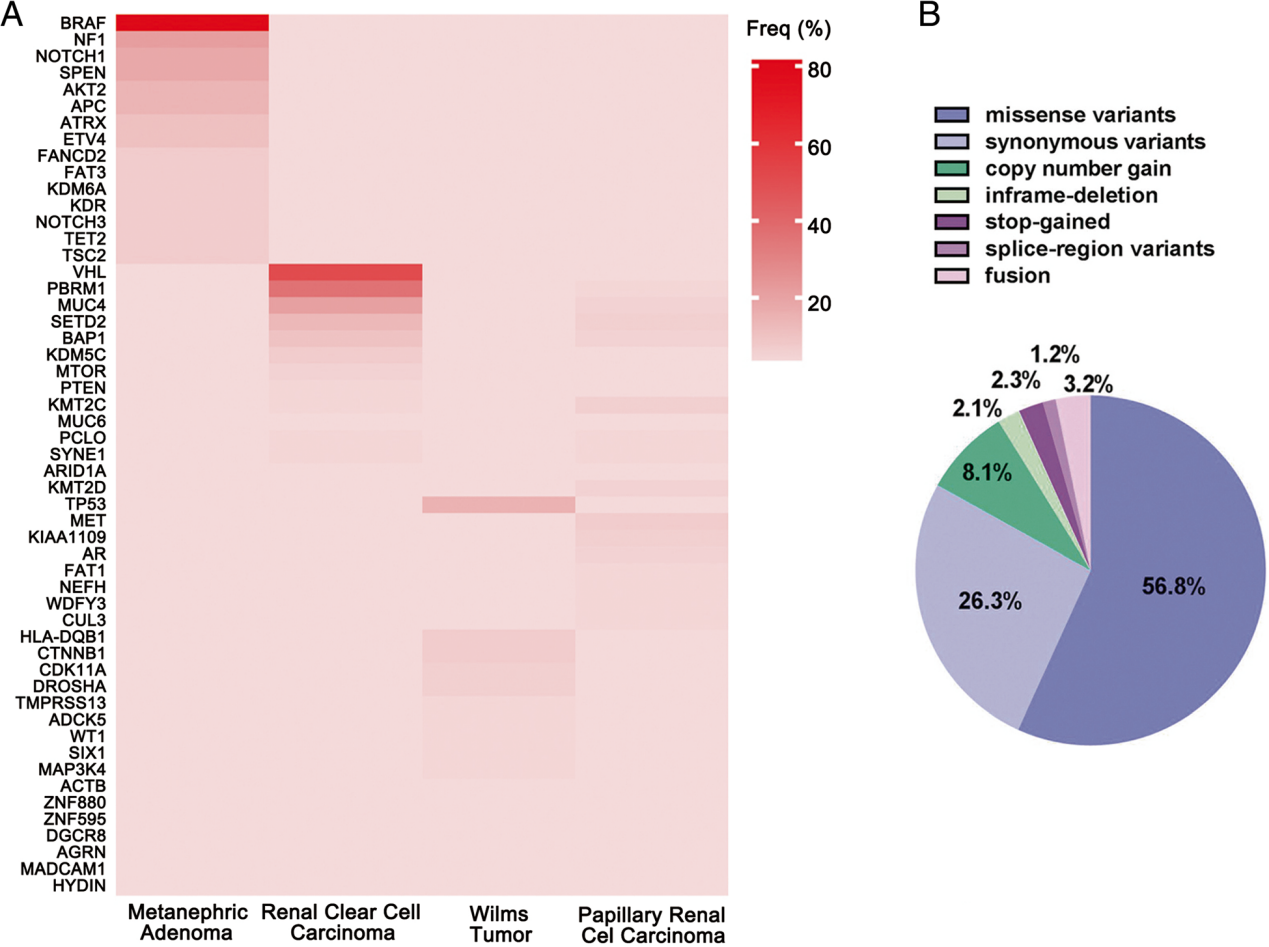
**a** The somatic mutation spectrum in different renal carcinoma. The hybrid capture-based NGS was used to sequence a panel of 295 well-established oncogene or oncosuppressors. In each analyzed MA patient, 85 somatic mutational events were detected. **b** The ratio of different mutant species in MA. The vast majority of mutation types were missense and synonymous variants (56.8 and 26.3%, respectively), while a smaller percentage was represented by copy number gain, indel, stop-gained, splice-region variants, or fusion (8.1, 2.1, 2.3, 1.2 and 3.2%, respectively)

To explore the genetic profile of MA, we used the hybrid capture-based NGS to sequence a panel of 295 well-established oncogene or oncosuppressors. In each analyzed MA patient, 85 somatic mutational events were detected. The vast majority of mutation types were missense and synonymous variants (56.8 and 26.3%, respectively), while a smaller percentage was represented by copy number gain, indel, stop-gained, splice-region variants, or fusion (8.1, 2.1, 2.3, 1.2 and 3.2%, respectively). In summary, the somatic mutation spectrum and mutation load (14.5/Mb) of MA was quite different from several other common types of renal carcinoma, such as renal clear cell carcinoma, Wilms tumor, and papillary renal cell carcinoma (Table 2, Fig. 2a).

**Table 2:** The somatic mutation spectrum in different renal carcinoma

	Present study (*n* = 28)			Renal Clear Cell Carcinoma (*n* = 499)			Wilms Tumor (*n* = 102)			Papillary Renal Cell Carcinoma (*n* = 293)		
	Gene	Mutant	Freq	Gene	Mutant	Freq	Gene	Mutant	Freq	Gene	Mutant	Freq
1	BRAF	23	82%	VHL	235	51%	TP53	15	15%	MET	23	7%
2	NF1	6	21%	PBRM1	158	36%	HLA-DQB1	11	7%	KMT2C	20	6%
3	NOTCH1	5	18%	MUC4	142	21%	CTNNB1	8	7%	MUC4	20	5%
4	SPEN	5	18%	SETD2	60	13%	CDK11A	6	6%	SETD2	19	6%
5	AKT2	4	14%	BAP1	44	10%	DROSHA	7	6%	KIAA1109	17	6%
6	APC	4	14%	KDM5C	30	7%	TMPRSS13	5	4%	BAP1	16	5%
7	ATRX	3	11%	MTOR	23	5%	ADCK5	4	4%	AR	16	5%
8	ETV4	3	11%	PABPC1	20	5%	WT1	4	4%	KMT2D	15	5%
9	FANCD2	2	7%	AHNAK2	19	4%	SIX1	4	4%	PCLO	14	4%
10	FAT3	2	7%	PTEN	19	4%	MAP3K4	4	4%	FAT1	13	4%
11	KDM6A	2	7%	ATM	18	3%	ACTB	3	3%	NEFH	13	4%
12	KDR	2	7%	KMT2C	17	4%	ZNF880	3	3%	WDFY3	13	4%
13	NOTCH3	2	7%	MAGEC1	16	4%	ZNF595	3	3%	SYNE1	13	4%
14	TET2	2	7%	MUC6	16	3%	DGCR8	3	3%	CUL3	13	4%
15	TSC2	2	7%	LRP1B	15	4%	AGRN	3	3%	ZNF814	13	3%
16				PCLO	15	4%	MADCAM1	4	3%	ALMS1	13	3%
17				SYNE1	15	4%	HYDIN	3	3%	DNAH8	12	4%
18				ARID1A	14	3%				CUBN	12	4%
19				KMT2D	14	3%				PBRM1	12	4%
20				MUC2	14	3%				PCF11	12	4%

It was shown that in MA, *BRAF* was the most frequently mutated gene (23 samples in a total of 28 MA cases, 82%), and *NF1* (6/28), *NOTCH1* (5/28), *SPEN* (5/28), *AKT2* (4/28), *APC* (4/28), *ATRX* (3/28), and *ETV4* (3/28) mutations were also frequently detected. Other gene mutations, such as *FANCD2*, *FAT3*, *KDM6A*, *KDR*, *TET2*, and *TSC2*, occurred twice in this MA cohort, which was quite different from other common types of renal carcinoma (Fig. 2b, Table 3). Meanwhile, some gene copy number gains, such as *AKT2*, *MET*, *ETV4*, *CCND1* and *FGFR1* were also detected in these MA samples (data not shown).

**Table 3:** Gene altered in MA

Gene	Number of mutation events (frequency)	Exon_Rank	Missense or stop-gained mutations	Base mutation	Synonymous or intronic mutations	Base mutation
BRAF	23	15	p.V600E, LOC100507389-BRAF, STARD9-BRAF, CUX1-BRAF	c.1799 T > A, chrom3:142634278_7:140492322, chrom15:42936216_7:140492968, chrom7:101809365_7:140492220	\	\
NF1	6	43, 46, 32	p.G1403S, p.Y2285*	c.4207G > A, c.6855C > A	p.S2152=	c.6456 T > C
NOTCH1	5	29, 32, 14	p.D1808N, p.H2018Q, p.D740N	c.5422G > A, c.6054C > A, c.2218G > A	\	\
SPEN	5	11, 2	p.R807H, p.R128C	c.2420G > A, c.382C > T	p.K926=	c.2778A > G
AKT2	4	NA	\		cn_amp	\
APC	4	16	p.L662I	c.1984C > A	p.P1634=	c.4902G > A
ATRX	3	9	\	\	p.T899=, p.S651=	c.2697G > A, c.1953 T > G
ETV4	3	NA	\	\	cn_amp	\
FANCD2	2	25, 30	p.N791S, p.T981I	c.2372A > G, c.2942C > T	\	\
FAT3	2	20, 23	p.R3849Q	c.11546G > A	p.Y4222=	c.12666C > T
KDR	2	10, 15	p.W460 L, p.I753V	c.1379G > T, c.2257A > G	\	\
NOTCH3	2	4, 24	p.A198V	c.593C > T	p.P1331=	c.3993G > A
TET2	2	11	p.E1973K, p.N1765 K	c.5917G > A, c.5295C > G	\	\
TSC2	2	30, 35, 5	p.V1144 M, p.E1513K	c.3430G > A, c.4537G > A	\	\
ABL1	1	4	\	\	p.L217=	c.649 T > C
ALOX12B	1	12	p.A525T	c.1573G > A	\	\
AMER1	1	2	p.G149_A156del	c.446_469delGAGCCACAGAGAAAGCTGTGGCTG	\	\
ARID1A	1	1	\	\	p.A281=	c.843G > A
ASXL1	1	12	p.Q778*	c.2332C > T	\	\
AURKB	1	6	p.G161R	c.481G > A	\	\
AXL	1	2	p.R71Q	c.212G > A	\	\
BCORL1	1	3	p.P312S	c.934C > T	\	\
CCND1	1	NA	\	\	cn_amp	\
CD79A	1	2	\	\	p.T65=	c.195C > T
CDH1	1	9	p.D433N	c.1297G > A	\	\
CDK12	1	13	\	\	p.S1191=	c.3573A > G
DIS3	1	11	p.V509 L	c.1525G > C	\	\
DOT1L	1	24	p.G1087S	c.3259G > A	\	\
EP300	1	2	\	\	p.Y207=	c.621C > T
EPHA3	1	7	p.A515T	c.1543G > A	\	\
EPHA5	1	5	\	\	p.Y437=	c.1311 T > C
ERBB2	1	3	\	\	p.P122=	c.366G > A
ERBB4	1	27	p.A1130S	c.3388G > T	\	\
EZH2	1	2	p.R18C	c.52C > T	\	\
FGFR1	1	NA	\	\	cn_amp	NA
FGFR4	1	18	p.D785H	c.2353G > C	\	\
GRIN2A	1	3	p.V122I	c.364G > A	\	\
HRAS	1	4	\	\	p.D108=	c.324C > T
IKBKE	1	6	p.R134C	c.400C > T	\	\
IL7R	1	5	p.A199G	c.596C > G	\	\
INHBA	1	2	p.K45R	c.134A > G	\	\
KAT6A	1	15	\	\	p.E993del	c.2977_2979delGAG
KDM5A	1	23	\	\	p.G1200 fs	c.3597dupA
KDM6A	1	5	p.Y143C	c.428A > G	\	\
KMT2D	1	10	\	\	p.P556=	c.1668G > T
MAP2K1	1	10	p.Q354H	c.1062A > C	\	\
MET	1	NA	\	\	cn_amp	NA
MTOR	1	28	\	\	p.A1388=	c.4164C > T
NCOR1	1	4	\	\	p.L133=	c.399G > A
NF2	1	5	\	\	p.R160=	c.478C > A
NPM1	1	7	p.V192 M	c.574G > A	\	\
NSD1	1	5	\	\	p.K513=	c.1539G > A
NTRK1	1	8	p.N323S	c.968A > G	\	\
PAK3	1	1	p.S31G	c.91A > G	\	\
PARP2	1	8	p.E231K	c.691G > A	\	\
PARP4	1	31	\	\	p.R1332=	c.3996C > T
PAX5	1	6	\	\	p.L234=	c.700C > T
PIK3C2G	1	29	p.R1316G	c.3946A > G	\	\
PIK3CG	1	8	\	\	p.I879=	c.2637C > T
PMS2	1	9	p.R304T	c.911G > C	\	\
PRDM1	1	5	p.H409Q	c.1227C > A	\	\
RAD52	1	9	p.A248T	c.742G > A	\	\
RET	1	7	\	\	p.G453=	c.1359G > C
RPTOR	1	14	\	\	p.N513=	c.1539C > T
SMARCA4	1	35	p.R1633Q	c.4898G > A	\	\
SOX10	1	4	p.T240P	c.718A > C	\	\
TSC1	1	13	p.M425 V	c.1273A > G	\	\
TSHR	1	5	p.G132R	c.394G > C	\	\

*Abbreviations*: *chrom* chromosome, *cn_amp* copy number gain amplification, *del* deletion

As previously reported, high frequency mutation of *BRAF* could be detected in MA [3]. In this study, we found 22 MA patients possessing a p.V600E mutation of *BRAF* exon 15, a substitution of thymidine by adenine (GTG → GAG) at codon 600. Meanwhile, a novel and rare *STARD9-BRAF*, *CUX1-BRAF* and *LOC100507389-BRAF* gene fusion was detected in one case. In this case, none of the other *BRAF* mutation variants was detected. All *BRAF* mutations have been confirmed using RT-PCR and Sanger sequencing (data not shown). In these BRAF V600E mutated MA patients (female: male = 16: 6), the median age was 40 years (ranging from 25 to 73 years) and the greatest dimension ranged from 2.5 to 7 cm (median = 3.2 cm). However, among the five cases with the wild-type *BRAF* gene, we found a striking gender difference (female: male = 1: 4), and the median age of BRAF wild-type patients tended to be 29 years (ranging from 12 to 47), which was smaller than BRAF V600E-mutated MA patients (*p* < 0.01). In addition, tumor size in BRAF wild-type cases ranged from 2 to 5.5 cm (median = 2.2); this value was also less than that in BRAF V600E-mutated patients (*p* < 0.05, Table 4). Although the morphological features were similar between MA and the other eight renal carcinoma cases which were initially misdiagnosed in this study, none of these renal carcinoma cases showed a *BRAF* exon 15 mutation (data not shown).

**Table 4:** Patient characteristics between BRAF V600E and BRAF wild-type MA patients

n	Total	BRAF V600E	BRAF wild-type
	27	22	5
Gender	F:M = 9:18	F:M = 5:17	F:M = 1:4*
Age range, year	12~ 80	25~ 73	12~ 47**
Median age, year	39	40	29
Tumor size, cm	2~ 7	2.5~ 7	2~ 5.5*
Median size, cm	3.1	3.2	2.2
BCL2, (negative/positive)	5/22	2/20	3/2**
p16, (negative/positive)	4/23	1/21	3/2**
p-MEK, (negative/positive)	0/27	0/22	0/5
p-ERK, (negative/positive)	15/12	13/9	2/3
DUSP6, (negative/positive)	4/23	2/20	2/3

*Abbreviations*: *F* female. *M* male. * *p* < 0.05, **p < 0.01 compared to BRAF V600E group. Statistical analysis was performed by Chi-square test

In addition, it should be noticed that one germline *BRCA1* (NM_007300.3) mutation which was likely pathogenic (c.2286A > T (p.Arg762Ser)) was found in a male MA patient. This patient also had a somatic BRAF V600E, APC L662I, and FANCD2 N791S missense mutation. He had no personal history of breast cancer, prostatic cancer, or pancreatic cancer, and no family history from his paternal or maternal branch was provided.

### Immunohistochemical analysis

**Fig. 3:**
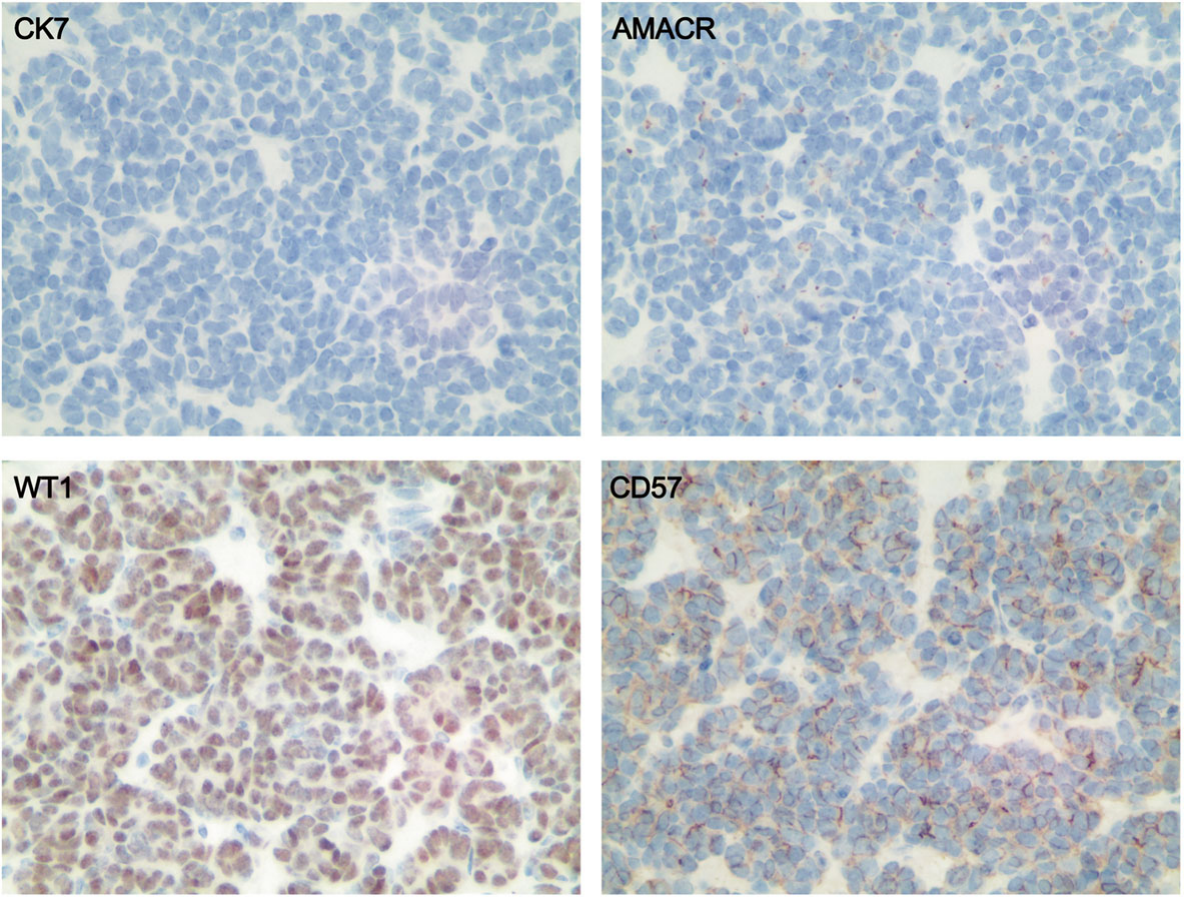
Immunohistochemical staining for pathological markers CK7, AMACR, WT1 and CD57 (400×) in representative MA tissues. The staining of positive tumor cells ranged from 1+ to 3+, indicating diffuse, moderate, and strong cytoplasmic expression

**Fig. 4:**
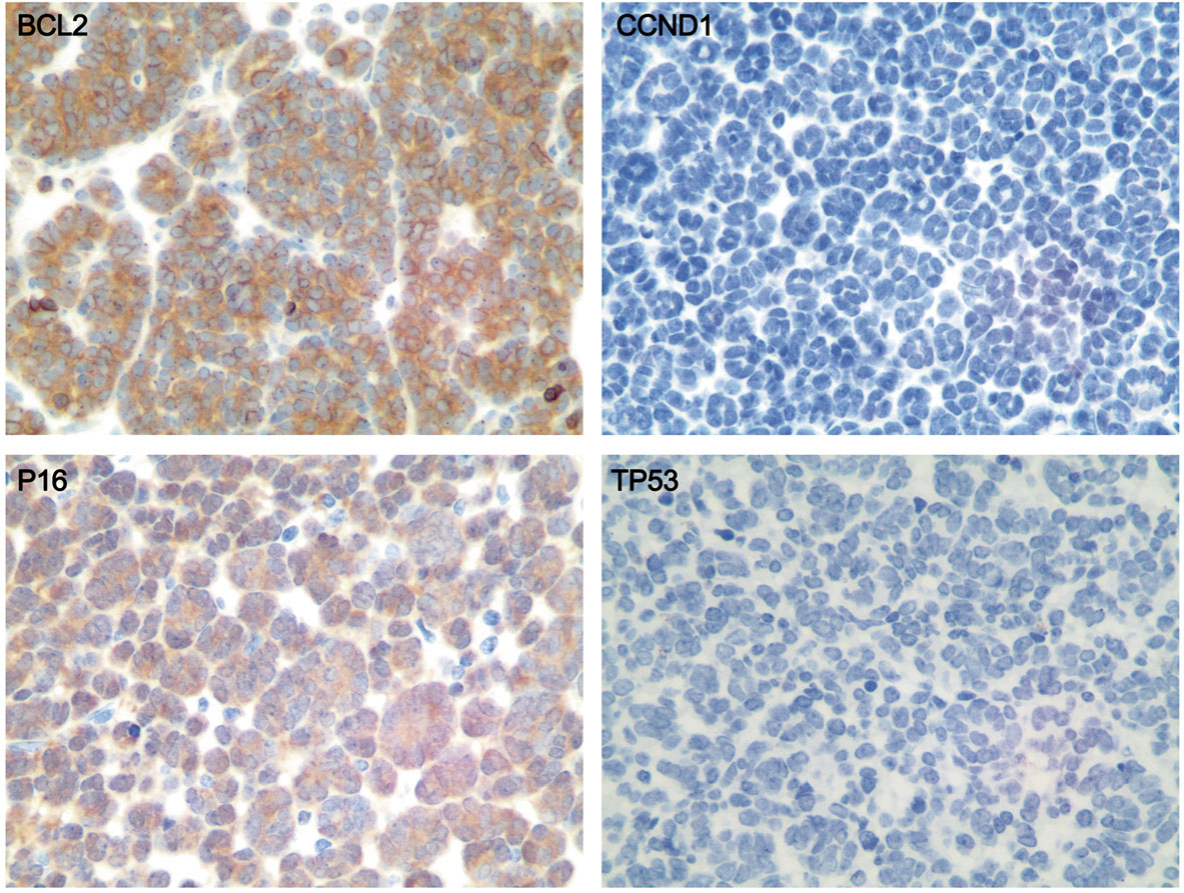
Immunohistochemical staining for BCL2, CCND1, P16 and TP53 in representative MA tissues, 400×. The staining of positive tumor cells ranged from 1+ to 3+, indicating diffuse, moderate, and strong cytoplasmic expression

Most of the MA tumors showed the expected staining pattern with negative CK7, AMACR, and positive WT1, CD57 (Fig. 3, Table 5). Only six cases (6/28, 21.4%) exhibited a discordant immunophenotype: two were focally positive for CK7 (CK7 +/−, AMACR -, WT1 +, and CD57 +), three were negative for WT1 (CK7 -, AMACR -, WT1 -, and CD57 +), and one was negative for CD57 (CK7 -, AMACR -, WT1 +, and CD57 -) (Table 5). Because MA is a slow growing tumor and rarely, if ever, exhibits aggressive behavior, we also analyzed specific markers that are closely associated with tumor cell growth and apoptosis, such as tumor suppressor TP53, anti-apoptosis protein BCL2, and cell-cycle related protein CCND1. Most of the MA cases exhibited the same staining pattern: TP53 -, BCL2 +, and CCND1 - (Fig. 4, Table 5). There were five cases (5/28, 17.9%) that showed different immunophenotypes (TP53 -, BCL2 -, and CCND1 -). These specific immunohistochemical results were summarized in Table 5. Meanwhile, in BRAF V600E-mutated MA, BCL2-positive cases were significantly higher than those in BRAF wild-type patients (Table 4).

**Table 5:** Immunohistochemical characteristics

Patient Number	BRAF status	CK7	AMACR	WT-1	CD57	P53	BCL2	CCND1	P16	p-ERK	ERK	p-MEK	MEK	DUSP6
1	BRAF V600E	–	–	3+	2+	–	3+	–	3+	–	3+	2+	3+	2+
2	BRAF wild-type	–	–	3+	3+	–	–	–	2+	–	3+	2+	3+	1+
3	BRAF V600E	–	–	1+	2+	–	–	–	1+	–	2+	1+	2+	1+
4	BRAF wild-type	–	–	2+	2+	–	–	–	1+	–	2+	1+	3+	–
5	BRAF V600E	–	–	3+	3+	–	1+	–	–	–	3+	2+	3+	1+
6	BRAF V600E	–	–	3+	2+	–	–	–	1+	–	2+	1+	2+	1+
7	BRAF V600E	–	–	1+	2+	–	2+	–	2+	1+	1+	1+	2+	1+
8	BRAF V600E	–	–	2+	2+	–	2+	–	2+	–	2+	1+	3+	–
9	BRAF V600E	–	–	3+	3+	–	3+	–	2+	1+	2+	1+	3+	1+
10	BRAF V600E	–	–	1+	3+	–	2+	–	2+	1+	1+	1+	3+	2+
11	BRAF V600E	–	–	3+	2+	–	3+	–	3+	1+	2+	1+	2+	2+
12	BRAF V600E	–	–	–	3+	–	3+	–	3+	–	2+	2+	1+	2+
13	BRAF V600E	–	–	2+	–	–	3+	–	2+	1+	1+	1+	2+	1+
14	BRAF V600E	–	–	3+	2+		2+	–	1+	1+	1+	1+	2+	1+
15	BRAF V600E	–	–	–	1+	–	3+	–	1+	–	2+	2+	1+	2+
16	BRAF V600E	–	–	1+	1+	–	2+	–	1+	–	1+	2+	3+	–
17	BRAF wild-type	–	–	1+	1+	–	–	–	–	1+	2+	1+	3+	1+
18	BRAF V600E	–	–	1+	1+	–	3+	–	3+	–	3+	1+	3+	1+
19	BRAF V600E	–	–	–	1+	–	3+	–	2+	–	3+	1+	1+	2+
20	BRAF Intron8-STARD9/ CUX1/ LOC100507389 fusion	+/−	–	1+	1+	–	3+	–	3+	1+	2+	1+	2+	1+
21	BRAF V600E	–	–	1+	1+	–	2+	–	1+	–	1+	1+	3+	1+
22	BRAF V600E	–	–	1+	1+	–	3+	–	1+	1	1+	1+	2+	1+
23	BRAF V600E	+/−	1+	1+	1+	–	3+	–	2+	1	1+	1+	2+	1+
24	BRAF wild-type	–	–	1+	1+	–	2+	–	–	1+	2+	1+	1+	1+
25	BRAF wild-type	–	–	1+	1+	–	3+	–	–	1+	3+	2+	2+	–
26	BRAF V600E	–	–	1+	1+	–	3+	–	1+	–	3+	1+	3+	2+
27	BRAF V600E	–	–	1+	1+	–	2+	–	2+	–	1+	1+	3+	1+
28	BRAF V600E	–	–	1+	1+	–	3+	–	1+	1+	1+	1+	1+	1+

It was reported that BRAF V600E induces senescence via regulation of p16 (INK4α) in some indolent or benign neoplasms [18]. Therefore, the expression of p16 (INK4α) protein in MA cases was also detected. It was shown that the vast majority of MA patients showed nuclear p16 (INK4α) immunoreactivity ranging from 1+ to 3+; only 4 cases showed negative or weak nuclear staining (Fig. 4, Table 5). Moreover, p16 (INK4α)-positive cases were significantly higher in BRAF V600E-mutated MA cases in comparison with those in the BRAF wild-type cases (Table 4).

**Fig. 5:**
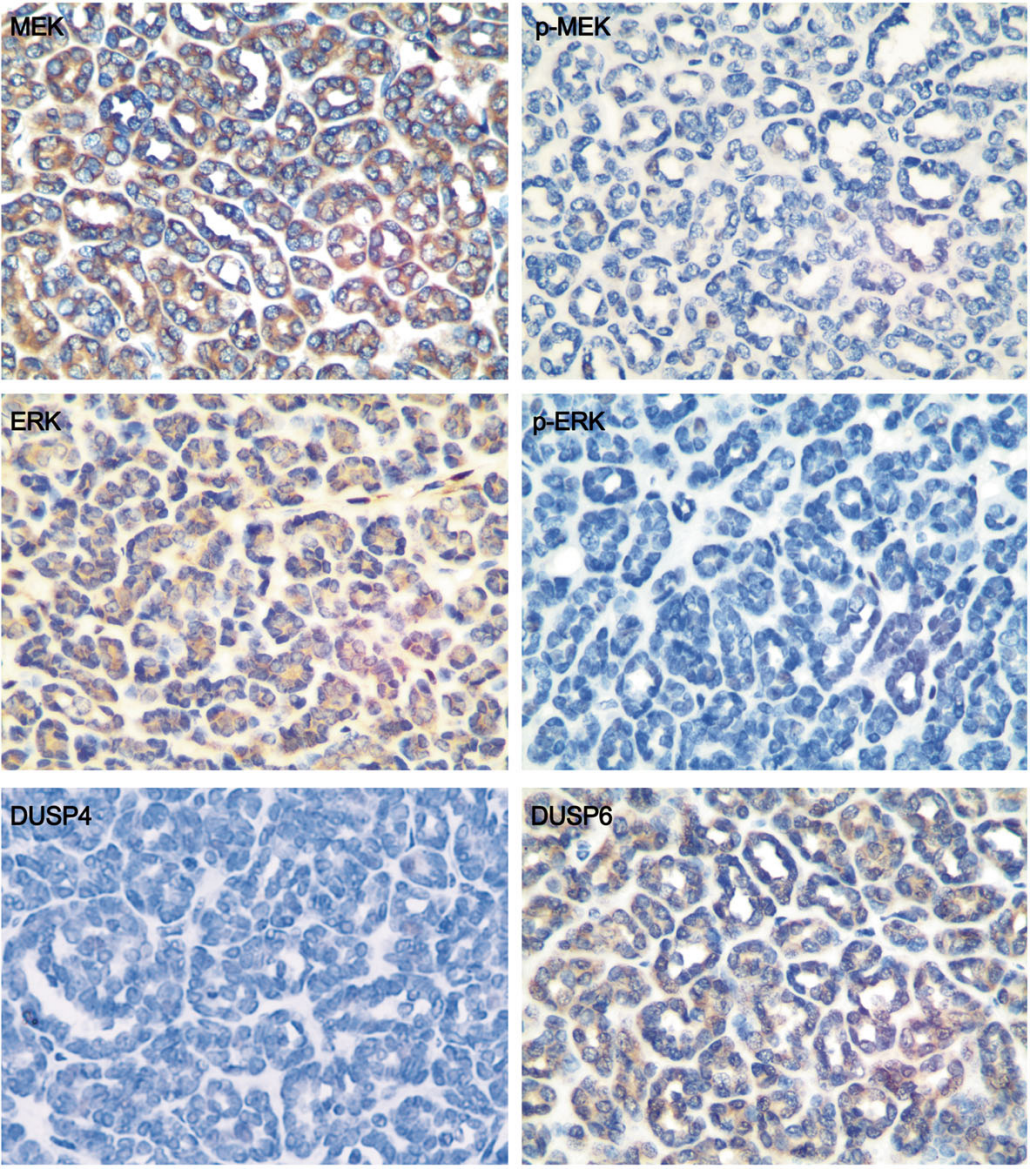
Immunohistochemical staining for MEK, p-MEK, ERK, p-ERK, DUSP4, DUSP6 in representative MA tissues, 400×. The staining of positive tumor cells ranged from 1+ to 3+, indicating diffuse, moderate, and strong cytoplasmic expression

Because of the high frequency of *BRAF* mutations in MA, we further investigated the status of MAPK signaling, the expression of phosphorylated MEK (p-MEK), total MEK (t-MEK), phosphorylated ERK (p-ERK), and total ERK (t-ERK). These were observed by immunostaining in the 28 MA cases, including 5 BRAF wild-type and 23 BRAF-mutated cases (Fig. 5, Table 5). Positive staining for p-MEK, t-MEK, and t-ERK was detected in the most MA cases. The staining of t-MEK and t-ERK positive tumor cells ranged from 1+ to 3+, indicating diffuse, moderate, and strong cytoplasmic expression. However, although p-MEK staining (ranged from 1+ to 2+) exhibited moderate nuclear staining in all 28 cases of MA, the nuclear staining for p-ERK was negative (15/28, 53.6%) or weakly (1+, 12/28, 42.9%) expressed among the different cases (Fig. 5, Table 5). In contrast to MA cells, morphologically normal kidney cortices adjacent to the tumors, including glomeruli, endothelial cells, and a subset of kidney tubules, demonstrated normally expressed and high levels of p-ERK, p-MEK, t-ERK and t-MEK (data not shown).

Suppressed ERK activation with normal MEK phosphorylation in MA drew our attention. Thus, some regulator genes which target ERK were investigated. It was found that except p16 (INK4α), a member of the DUSPs superfamily, DUSP6 was diffusely expressed in most of the MA cases, ranging from 1+ to 3+ (Fig. 5, Table 5). However, no significant difference in p-ERK nor DUSP6 was found between BRAF V600E-mutated and BRAF wild-type MA cases (Table 5). Besides, less DUSP6 staining was detected in the morphologically normal kidney cortex adjacent to the tumors and other ERK regulators such as DUSP4 (Fig. 5), PTPRR and p62 (data not shown), which were less expressed in the MA cells.

## Discussion

It is known that there can be similar histopathological features between MA and some other renal tumors [5, 19]. Thus, novel genetic analysis, including BRAF V600E mutation in MA, could improve the diagnosis of this benign renal tumor. However, except for approximately 10% of the wild-type genotype, other BRAF mutation sites such as V600D, V600 K, V600R, and K601 L also exist in MA patients [6, 20]. We further analyzed those *BRAF* wild-type cases of MA. All five cases of MA which had negative *BRAF* mutation showed typical morphologic features of MA. Meanwhile, their immunophenotyping were WT-1 +, CD57 +, CK7 -, AMACR -, which were also accorded with the diagnosis of MA [6]. Moreover, these patients received good treatment outcomes and good prognosis, which differs them from malignant renal tumors. We also detected copy number variations of 7, 17 and Y chromosomes by FISH technology, and found no abnormities among these five cases of MA, which is characteristic of type 1 PRCC (data not shown). All these morphologic features, immunohistochemical patterns, clinical characteristics and long-term prognosis supported that although the *BRAF* mutation were negative, these five cases still should be identified as MA. However, although negative *BRAF* exists in MA, considered the high frequency of *BRAF* mutation improves the diagnosis of MA. It should be noticed that metanephric stromal tumors (MST), another benign renal tumor, and the BRAF V600E mutation were frequently detected [21, 22]. Therefore, finding more pathological and molecular markers is still essential for improving the diagnosis of MA.

In this study, we examined the genetic profiles of MA in a large cohort of Chinese MA patients from multiple pathology centers using NGS-based gene analysis for the first time. Similar to previous studies mainly conducted on Caucasian MA patients [3, 20, 23], it was found that MA patients from Asian populations also have a high rate of mutation in the BRAF V600E site. This finding can further improve the using of BRAF V600E detection in the diagnosis of MA. It should be noticed that a novel *STARD9*-*BRAF*, *CUX1*-*BRAF*, and *LOC100507389*-*BRAF* gene fusion was found at *BRAF* intron 8 without V600E mutation. As MA is a rare, benign renal tumor, the clinical significance of this novel *BRAF* gene fusion remains unknown. However, recent studies showed that different *BRAF* fusions, such as *GTF2I*-*BRAF* [24], *DGKI*-*BRAF* [25], and *TMEM106B*-*BRAF* [26], activated the MAPK pathway, thereby regulating tumor growth in multiple cancers. Therefore, it could be suspected that this novel three-sites *BRAF* gene fusion also intervenes with the MAPK pathway, which may play an important role in not only MA but also in other malignant tumors.

Using NGS analysis, not only the BRAF V600E mutation, but also other somatic mutations, including *NF1*, *NOTCH1*, *SPEN*, *AKT2*, *APC*, *ATRX*, and *ETV4*, were found to have a high mutation rate in MA. Thus, the somatic mutation spectrum of MA could be described, which was quite different from several other renal tumors such as renal clear cell carcinoma, renal non-clear cell carcinoma, and papillary renal cell carcinoma. It was known that the histopathological diagnosis of MA faced challenges [19]. In our total thirty-six cases originally diagnosed as MA, eight of them were misdiagnosed. Although these misdiagnosed cases from other participating institutions may be caused by different diagnostic level, similar morphological between MA and other renal carcinomas like Wilms tumor or PRCC increased the difficulty of diagnosis. Thus, clarifying the somatic mutation spectrum could improve the identification of MA in pathological diagnosis, especially in some difficult cases. Although it was confirmed that there was no copy number gain in chromosomes 3, 7, 17, and Y in those 28 MA cases by FISH analysis (data not shown), which is frequently existed in type 1 PRCC [8], NGS results still suggested that approximately 8.1% copy number gain occurred in total mutations. As it was known, the copy number variants were highly associated with the development of malignancy, such as type 1 PRCC [27]. It was reported that in some cases, which arose as Wilms tumor or PRCC, might develop to be similar in morphology with MA, thereby leading to misdiagnosis [28]. Therefore, improving the diagnosis of MA would be important in the prognosis of patients, and the effect of detected copy number variants in MA should be further identified.

Interestingly, a rare germline *BRCA1* mutation was found in one male MA patient without a personal or family history of breast cancer, prostatic cancer, or pancreatic cancer. It was widely known that *BRCA1/2* are important tumor suppressor genes, which help DNA repair and promote cell apoptosis in breast and other tissues [29]. And certain mutations of *BRCA1/2* often lead to increasing the risk of breast and ovarian cancer in women [30]. However, *BRCA1* mutation was less investigated in MA and other renal carcinomas. After surgical removal, this MA patient was still alive, and no other tumors were found. Because this sole case cannot establish the relationship between this germline *BRCA1* mutation and tumor development as MA, the role of this famous tumor suppressor gene in MA requires further evaluation.

Based on the genetic profile of MA, we further investigated the expression of some molecular markers associated with tumor cell survival and the MAPK pathway. Similar to previous studies, we found that MA patients had high rates of BRAF V600E mutation. It was known that BRAF V600E could sustain the activation of its downstream kinase MEK in the MAPK pathway, thereby stimulating cell division and differentiation in some malignancies [31]. Thus, as well as in other indolent neoplasms such as melanocytic nevi [12], the reason for why MA remains benign in the presence of a BRAF V600E mutation attracted our attention. One theory partly explained that the BRAF V600E mutation could lead to cell arrest via induction of p16 (INK4α). The well-known tumor suppressor p16 (INK4α), encoded by the human *CDKN2A* gene, could inhibit the cell cycle from the G1 to the S phase, thereby decelerating tumor cell division and leading to cell senescence [32]. In human melanocytes, sustained BRAF V600E mutation could induce cell cycle arrest accompanied by immunoreactivity of p16 (INK4α) [18]. Meanwhile, BRAF V600E expression stopped proliferation and induced markers of oncogene-induced senescence including p16 (INK4α) in human neural stem cells [33]. Choueiri et al. reported that p-MEK and p-ERK were both positive in BRAF V600E-mutated or wild-type MA patients with the p16 (INK4α) senescence marker [3]. In this study, we confirmed that p16 (INK4α) was highly expressed in most MA patients, which may be important in maintaining MA as an indolent tumor. Differently, our data showed higher p16 (INK4α)-positive rates in BRAF V600E-mutated MA than in the BRAF wild-type, which increased the relationship between BRAF V600E and the p16 (INK4α) senescence marker in those indolent neoplasms. While the human *CDKN2A* gene is mutated or deleted, which frequently occurred in some malignancies such as melanoma, tumor cells would overcome BRAF V600E-induced senescence and become malignant [34]. However, the development patterns in indolent neoplasms should be complex. Not only p16 (INK4α), but other senescence markers such as acidic β-galactosidase and PAI-1, could also be involved in cell senescence induced by a *BRAF* mutation [14].

Moreover, factors that directly affect the MAPK pathway may also participate in the development of indolent neoplasms. Kim et al. found that ERK phosphorylation was not increased in BRAF V600E-mutated papillary thyroid carcinomas; conversely, phosphorylation decreased, even compared to normal thyroid glands [35]. Therefore, although p-ERK positivity was observed in MA in a previous study, to further investigate the status of the MAPK pathway in MA, we verified MEK and ERK phosphorylation by immunostaining. As we expected, MEK kinase was phosphorylated in both *BRAF*-mutated and wild-type cases. But only 44.4% of MA cases showed a low grade of p-ERK positivity. In the rest of the cases, the immunoreactivity of p-ERK was weak or entirely negative. Besides, the p-ERK positivity was no different between the *BRAF*-mutated and wild-type MA cases, suggesting that a *BRAF* mutation causes an irrelevant pathway that blocks the MAPK signal, which may be another important reason for the indolence of MA.

**Fig. 6:**
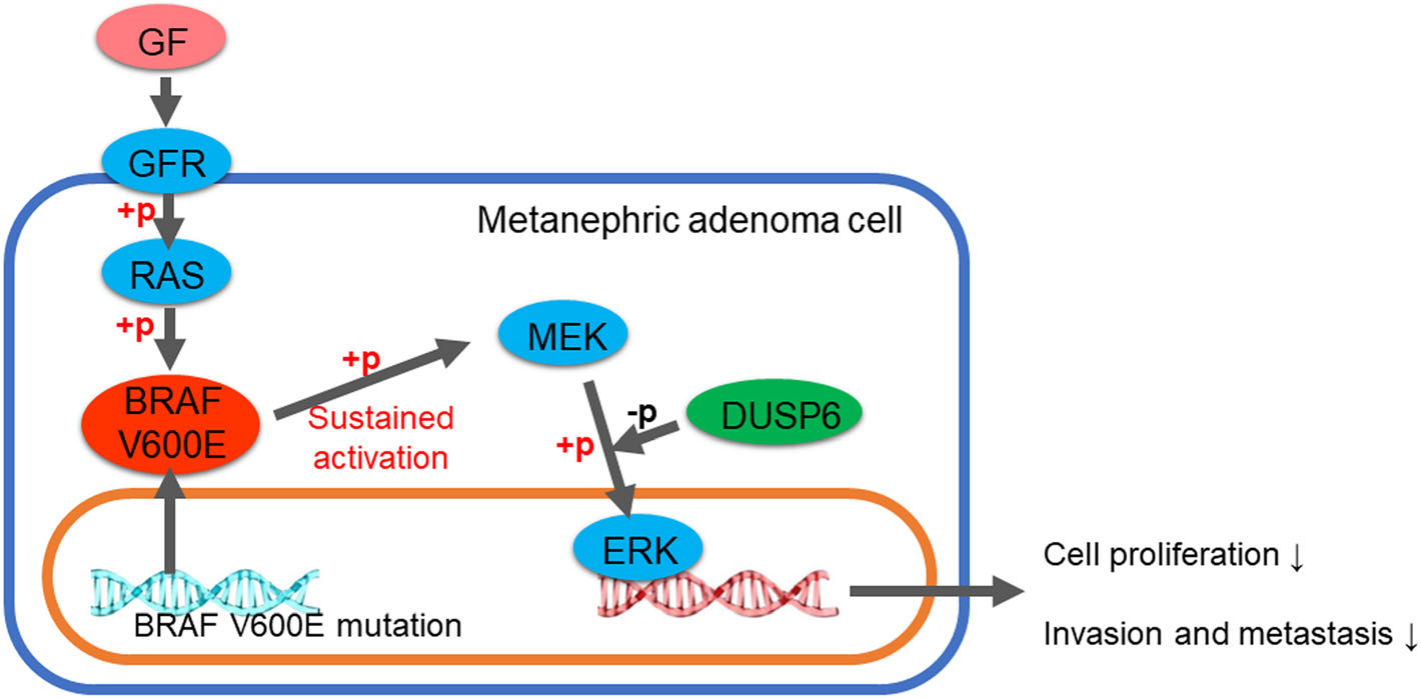
Pathogenesis of metanephric adenoma

Signals that directly inhibit ERK phosphorylation may explain this phenomenon. It has been reported that the up-regulated expression of MAP phosphatase 3/dual specificity phosphatase family of protein 6 (MKP3/DUSP6), a cytosolic ERK1/2-targeted phosphatase, contributed to the senescence of NRK-52E rat renal tubular epithelial cells via dephosphorylation of ERK1/2 [36]. Meanwhile, DUSP4/MKP2 overexpression is also associated with the aggressive behavior of BRAF V600E-mutated papillary thyroid cancer [37]. Therefore, the expression of ERK1/2 phosphatases DUSP4 and DUSP6 were determined by immunohistochemistry. It was found that DUSP6 was highly expressed, while DUSP4 was less positive in most MA cases. Although the deficiency of MA case number limited the research on the relationship between DUSPs and BRAF V600E mutation, and it was difficult to perform an in vitro experiment for MA, we could still speculate that direct regulation on ERK1/2 phosphorylation by DUSP6 plays an important role in the indolent behavior of MA (Fig. 6).

## Conclusions

In this study, we investigated the genetic profile of MA samples based on a large retrospective cohort of the Asian population for the first time. The establishment of the somatic mutation spectrum could contribute to the diagnosis of MA and other potentially misdiagnosed renal carcinomas. Meanwhile, based on genetic analysis, we suggested that DUSP6-induced ERK1/2 dephosphorylation could be important for the indolent behavior of MA and could also be identified as a potential diagnostic marker for MA. Both novel clinicopathological and molecular features could provide benefits for the diagnosis and better understanding of this rare, potentially misdiagnosed benign renal tumor.

## Additional files

Additional file 1: 295 genes in capture-based targeted sequencing panel. (DOCX 16 kb)

Additional file 2: Patient characteristics. (DOCX 16 kb)

## Abbreviations

FFPE: Formalin fixed paraffin-embedded
FISH: Fluorescence in situ hybridization
H&E: Hematoxylin and eosin
MA: Metanephric adenoma
MAPK: Mitogen-activated protein kinase
MKP3/DUSP6: MAP phosphatase 3/dual specificity phosphatase family of protein 6
MTSCC: Mucinous tubular and spindle cell carcinoma
NGS: Next-Generation Sequencing
PRCC: Papillary renal cell carcinoma
