# Supplementary material for: Novel clinicopathological and molecular characterization of metanephric adenoma: a study of 28 cases
Source: Diagn Pathol. 2018 Aug 16;13:54. doi: 10.1186/s13000-018-0732-x (PMC6094885; doi:10.1186/s13000-018-0732-x)
Supplement: Supplementary file 1 — 295 genes in capture-based targeted sequencing panel. (DOCX 16 kb) [file 13000_2018_732_MOESM1_ESM.docx]

Supplementary Table 1. 295 genes in capture-based targeted sequencing panel

| Mutation type | Genes |
| --- | --- |
| Mutation | ABL1; AKT1; AKT2; AKT3; ALOX12B; AMER1; APC; APCDD1; ARAF; ARID1A; ARID2; ASXL1; ATM; ATR; ATRX; AXL; BACH1; BAP1; BARD1; BCL2L2; BCOR; BCORL1; BLM; BRAF; BRCA2; BRIP1; BTK; CARD11; CASP8; CBFB; CBL; CDH1; CHEK1; CHEK2; CHUK; CIC; CRBN; CREBBP; CSF1R; CTCF; CTNNA1; CTNNB1; CUL4B; CYP17A1; DAXX; DIS3; DNMT3A; DOT1L; EP300; EPHA3; EPHA5; EPHB1; ERBB3; ERBB4; ERG; EZH2; FAM46C; FANCA; FANCC; FANCD2; FANCE; FANCF; FANCG; FANCI; FANCL; FANCM; FAT3; FBXW7; FGFR4; FLT1; FLT3; FLT4; GATA1; GATA2; GATA3; GNA11; GNA13; GNAQ; GNAS; GRIN2A; GSK3B; HGF; HLA-A; HRAF; IDH1; IDH2; IGF1; IGF1R; IGF2; IL7R; INHBA; JAK1; JAK2; JAK3; KAT6A; KDM5A; KDM5C; KDM6A; KDR; KEAP1; KIT; KMT2A; KMT2D; KRAS; LRP1B; MAP2K1; MAP2K2; MAP2K4; MAP3K1; MAP3K13; MDM2; MDM2; MED12; MEN1; MLH1; MPL; MRE11A; MSH2; MSH6; MTOR; MUTYH; MYD88; NCOR1; NF1; NF2; NFE2L2; NOTCH1; NOTCH2; NOTCH3; NOTCH4; NPM1; NRAS; NSD1; NTRK2; NUP93; PAK3; PAK7; PALB2; PAX5; PBRM1; PDGFRA; PDGFRB; PDK1; PIK3C2G; PIK3C3; PIK3CA; PIK3CG; PIK3R1; PIK3R2; PMS2; PPP2R1A; PRDM1; PRKAR1A; PRKDC; PTCH1; PTEN; PTPN11; RAD50; RAD51; RAD51B; RAD51C; RAD51D; RAD52; RAD54L; RAF1; RB1; RICTOR; RNF43; RPA1; RPTOR; RUNX1; RUNX1T1; SETD2; SF3B1; SH2B3; SMAD2; SMAD4; SMARCA4; SMARCB1; SMARCD1; SMO; SOCS1; SOX10; SOX2; SPEN; SPOP; SRC; STAG2; STAT4; STK11; SUFU; SYK; TBX3; TET2; TGFBR2; TNFAIP3; TOP1; TP53; TRRAP; TSC1; TSC2; TSHR; VHL; WISP3; WT1; XPO1 |
| Mutation  /Amplification | AR; ARFRP1; AURKA; AURKB; BCL6; BTG1; C11ORF30; CCND1; CCND2; CCND3; CCNE1; CD79A; CD79B; CDC73; CDK12; CDK4; CDK6; CDK8; CDKN1B; CDKN2A; CDKN2B; CDKN2C; CEBPA; CRKL; CRLF2; CUL4A; DDR2; EGFR; ERBB2; ESR1; FGF10; FGF12; FGF14; FGF19; FGF23; FGF3; FGF4; FGF6; FGF7; FGFR1; FGFR2; FGFR3; FOXL2; GID4; GPR124; IKBKE; IKZF1; IFR4; IRS2; JUN; KLHL6; LMO1; MCL1; MEF2B; MET; MYC; MYCL1; MYCN; NBN; NFKBIA; NKX2-1; PARP1; PARP2; PARP3; PARP4; PNRC1; PRSS8; REL; TIPARP; TNFRSF14; XRCC3; ZNF217; ZNF703 |
| Mutation  /Fusion | ALK; BCL2; BCR; BRCA1; ETV1; ETV4; ETV5; ETV6; EWSR1; FGFR1; FGFR2; FGFR3; NTRK1; NTRK3; RARA; RET; ROS1; TMPRSS2 |
