# Supplementary material for: Novel clinicopathological and molecular characterization of metanephric adenoma: a study of 28 cases
Source: Diagn Pathol. 2018 Aug 16;13:54. doi: 10.1186/s13000-018-0732-x (PMC6094885; doi:10.1186/s13000-018-0732-x)
Supplement: Supplementary file 2 — Patient characteristics. (DOCX 16 kb) [file 13000_2018_732_MOESM2_ESM.docx]

Supplementary Table 2. Patient characteristics

| Patient No. | Gender | Tumor size, cm | Patient No. | Gender | Tumor size, cm |
| --- | --- | --- | --- | --- | --- |
| 1 | F | 4.5 | **15** | M | 5.5 |
| 2 | F | 3 | **16** | F | 4.5 |
| 3 | F | 2.5 | **17** | M | 7 |
| 4 | M | 3 | **18** | F | 5 |
| 5 | M | 2.5 | **19** | F | 4.5 |
| 6 | M | 3.2 | **20** | F | 2.5 |
| 7 | F | 17 | **21** | M | 5 |
| 8 | F | 3 | **22** | F | 5 |
| 9 | M | 3 | **23** | F | 5 |
| 10 | F | 3.5 | **24** | M | 3.5 |
| 11 | F | 3 | **25** | M | 2 |
| 12 | F | 3.2 | **26** | F | 2 |
| 13 | F | 2.6 | **27** | M | 5.5 |
| 14 | F | 6 | **28** | F | 2.2 |
